# Genetic and Environmental Factors in Autoimmune Thyroid Disease: Exploring Associations with Selenium Levels and Novel Loci in a Latvian Cohort

**DOI:** 10.3390/cimb46030162

**Published:** 2024-03-17

**Authors:** Sabine Upmale-Engela, Ieva Vaivode, Raitis Peculis, Helena Litvina, Tatjana Zake, Andrejs Skesters, Deniss Gogins, Vita Rovite, Ilze Konrade

**Affiliations:** 1 Department of Internal Medicine, Riga Stradins University, LV-1007 Riga, Latvia, , ,; 2 Latvian Biomedical Research and Study Centre, Ratsupites Str. 1-k1, LV-1067 Riga, Latvia, ,; 3 Laboratory of Biochemistry, Riga Stradins University, LV-1007 Riga, Latvia; 4 Department of Endocrinology, Riga East Clinical Hospital, Hipokrata Str. 2, LV-1038 Riga, Latvia

**Keywords:** autoimmune thyroid diseases, genome-wide association, selenium

## Abstract

The interplay of genetic, immune and environmental factors strongly contributes to the development of autoimmune thyroid disease (AITD), which can be classified as Graves’ disease (GD) or Hashimoto thyroiditis (HT). One of the most studied exogenous factors in the pathogenesis of AITD is selenium, which, in the form of selenoproteins, strengthens the antioxidative defence system of thyroid cells against superoxide production. Furthermore, it modulates inflammatory cytokine release and autoantibody production. The aim of this study was to assess the associations of genetic factors with selenium levels in a cohort of adults with HT and GD and healthy controls from Latvia. A total of 148 GD patients, 102 HT patients and 2442 control participants were included in the study. The genotypes were determined using genome-wide genotyping; imputation was carried out using the TOPMed r2 imputation panel; and association analysis was performed with PLINK v1.90b7. We found three loci associated with GD (LSAMP, HNRNPA3P5, and NTN1) and one locus associated with HT (VAT1L); furthermore, one locus was associated with a serum selenium concentration > 80 µg/L (LINC01544/RNF152/PIGN). The detected associations could be attributed to population-specific effects or unknown stratification in our cohort, and further assessment of these results is required to explain the relationships of genetic traits with AITD and other phenotypes.

## 1. Introduction

Autoimmune thyroid disease (AITD) is one of the most common autoimmune diseases (ADs) [1], with an estimated prevalence of at least 5% in the general population [2]. AITD comprises two main interrelated conditions, Graves’ disease (GD) and Hashimoto’s thyroiditis (HT), which mainly manifest as thyrotoxicosis and hypothyroidism, respectively [3]. Despite GD and HT having similar characteristics, e.g., lymphocytic infiltration of the thyroid and increased levels of thyroid autoantibodies, each disease exhibits a distinct immunopathogenesis [4,5]. This finding suggests the participation of immune-modulating genes in the pathogenesis of AITD [4]. Associations with several immune-related genes, other than HLA genes, have been described in a number of autoimmune diseases and allegedly underpin the genetically determined susceptibility to autoimmunity [1]. Moreover, the pathogenesis of AITD involves immune-related genes, and approximately 80% of the susceptibility to GD development is caused by the presence of specific alleles of certain genes [6]. These genes include regulatory T-cell genes (e.g., *FOXP3 and CD25*), immunological synapse genes [e.g., *HLA-DR*, *CTLA-4*, and *CD40*], and the protein tyrosine phosphatase, nonreceptor type 22 (*PTPN22*) gene [7]. Predisposition to both GD and HT is associated with polymorphisms in specific alleles of *CTLA-4* [1].

GWASs have suggested that there are more than 20 genes associated with susceptibility to GD; however, only 20% of the genetic causes of GD can be attributed to these variations. This necessitates the need to screen for additional genetic variations, including rare variants and copy number variations [8]. Additionally, approximately 5.5% of the genetic factors predisposing patients to HT have been identified; therefore, this topic remains poorly understood [9].

Furthermore, a combination of genetic, immune, environmental, and alimentary factors plays a pivotal role in the development of AITD [10]. Selenium is an essential micronutrient for thyroid hormone metabolism and proper antioxidant function; therefore, selenium supplementation is thought to improve the clinical features of AITD [11]. Genetic variations can affect the absorption, distribution and excretion of essential trace elements, including selenium. We tested the impact of single-nucleotide polymorphisms (SNPs) on selenium levels and the associations with AITD in a genome-wide association study (GWAS) using a cohort of adults with HT and GD and healthy controls from Latvia.

## 2. Materials and Methods

### 2.1. Study Design

This study includes three distinct parts to investigate connection between genotype, thyroid disease and selenium levels in the Latvian population. First, we used a cohort from the national biobank Genome Database of Latvian Population [12] consisting of 148 GD patients, 102 HT patients and 2442 controls for GWAS of AITD in Latvia. This cohort includes both retrospective samples collected over time from 135 GD patients and 55 HT patients as well as controls, which in this study include 2421 biobank participants representing the general population and the disease-specific biobank participants with available genome-wide genotyping data. Additionally, 13 GD patients, 47 HT patients, and 21 individuals in the control group were included for genotyping once the study started.

Second, we performed a literature search to identify genes and SNPs previously associated with AITD and serum selenium. The results were used to check whether associations are present in the Latvian population.

Third, all newly recruited individuals and retrospective participants with serum samples in biobank (with an emphasis on AITD patients) were selected for serum selenium measurements and selenium-level GWAS in dichotomous case–control type analysis using a > 80 µg/L threshold to distinguish between low and reference selenium levels.

### 2.2. Clinical and Biochemical Data of the Study Cohort

All enrolled participants underwent thyroid function testing. The diagnosis of GD was confirmed if the thyroid-stimulating hormone (TSH) level was below the reference range (0.35–4.94 μIU/mL) and if positive thyroid receptor antibodies were detected (reference range: 0–1.58 IU/L). HT was confirmed if anti-thyroid peroxidase (anti-TPO) antibody or anti-thyroglobulin (anti-Tg) antibody levels were at least 1.5 times above the upper limit of the reference range (0–5.61 IU/mL and 0–40 U/mL, respectively). The diagnosis of AITD was established by a physician through an assessment of the clinical symptoms associated with Graves’ disease (GD) and Hashimoto’s thyroiditis (HT), along with the results of biochemical thyroid tests and ultrasound imaging, evaluating thyroid size, echotexture and vascularization. The exclusion criteria for the study were pregnancy, malignancy, active infection, other known autoimmune diseases, renal or hepatic impairment, and the use of selenium-containing supplements or medications that can affect thyroid function (glucocorticoids, anticonvulsants, antidepressants, amiodarone).

### 2.3. Assessment of Selenium Levels

The serum selenium concentration was measured with a “Cary Eclipse” fluorescence spectrophotometer (Varian, Inc., Houten, The Netherlands). Interlaboratory quality control was implemented by two standards, Selenium AAS (Aldrich, St. Louis, MO, USA; Cat. No. 24,792-8) and Seronorm TE Serum Level I (Sero AS; Cat. No. 201 405, Billingstad, Norway) from the Seronorm™ Trace Elements-Controls Programme. Labquality Oy, Helsinki, Finland, provided external quality assessment services. The reference range for selenium levels was 80–125 μg/L [13,14].

### 2.4. Assessment of Thyroid Function and Autoimmunity

Thyroid function was assessed by measuring serum TSH, free thyroxine (FT4), and (free triiodothyronine) FT3 levels. In addition, anti-thyroid peroxidase (anti-TPO) and anti-thyroglobulin (anti-Tg) antibodies were detected via chemiluminescence immunoassay (Siemens) performed on an Advia Centaur XP (Siemens Healthcare Diagnostics, Inc., Tarrytown, NY, USA) analyser. Additionally, TSH receptor antibodies (TRAbs), anti-nuclear antibodies (ANAs), and tissue transglutaminase IgA (tTG-IgA) antibodies were detected via ELISA (Pharmacia Diagnostics, Freiburg, Germany) according to the manufacturer’s instructions.

### 2.5. Genotyping

DNA was isolated from whole-blood samples using the phenol–chloroform extraction method in the biobank setting as previously described [12]. DNA samples were quantified with a Qubit^®^ 2.0 fluorometer using a Qubit dsDNA HS Assay Kit (Thermo Fisher Scientific, Waltham, MA, USA). DNA samples were diluted into 96-well PCR plates to a final concentration of 28 ng per well using a Freedom Evo robotic workstation (Tecan, Männedorf, Switzerland) with disposable filter tips. The samples were genotyped on an Infinium Global Screening Array, Affymetrix Axiom Genome-Wide Human EU and OmniExpress Exome genotyping array. Patients were genotyped in a single batch using the Infinium Global Screening Array genotyping chip. The controls were genotyped in six separate batches with all three genotyping arrays.

### 2.6. Association Analysis

Genotype quality control was based on the work of Lam et al. 2020 [15], and imputation was carried out using the TOPMed r2 imputation panel in the TOPMed imputation server [16]. Association analysis was performed with PLINK v1.90b7 [17] for selenium levels and SAIGE v1.0.0 software for disease association [18]. We assessed the associations between genotypes and the following phenotypes using case–control design: selenium concentration (>80 μg/L designated as case, <80 μg/L designated as controls), presence of Graves’ disease and presence of Hashimoto thyroiditis. The sex, age and batch number of the genotyped participants were added as covariates in all analyses. Status of Graves’ disease and Hashimoto thyroiditis was included as a covariate in the analysis of selenium concentration. To control for population stratification, we used the first 10 principal components. A high case-to-control ratio was used to increase the study power because of the relatively small number of cases [19]. Manhattan plot visualisation was performed with the LocusZoom interactive platform [20]. To determine the functional role of allelic variants, expression quantitative trait locus (eQTL) analysis was conducted by using the open-access Genotype Tissue Expression (GTEx) database [21].

### 2.7. Study Power Calculation

We performed post hoc study power calculation with Quanto v1.2.4 software based on the obtained results and allele frequencies in the Non-Finnish European population. Additional parameters included unmatched case–control design with 16.5 controls per case (using our *n* = 148) and a gene-only hypothesis with dominant action of SNP minor alleles. The population estimated risks for thyroid disease were set to 0.5%. A two-sided test was used. SNPs from chromosomes 3, 13 and 16 had over 80% power to detect the observed genetic effect in 95% confidence intervals, while SNPs from chromosome 17 (rs189272113) had 80% power to detect the genetic risk of about 4.45. When the case–control ratio was set to 1, the two rarest SNPs (chr3 rs147639537 at 0.9% and chr17 rs189272113 at 0.5%) did not demonstrate statistical power in the lower half of the 95% confidence interval.

As for the prospective selenium-level GWAS, 80% study power was reached at a genetic risk of 2.2, leaving only the lowest part of the confidence interval of 95% underpowered. Assumptions for this calculation are as follows: unmatched case–control design (0.75 controls per case), allele frequency 50%, risk 50%, dominant gene only hypothesis and two-sided test.

## 3. Results

### 3.1. Characterisation of the AITD Study Cohort

A summary of the characteristics of the study cohort, including 148 GD patients, 102 HT patients and 2442 control participants, is shown in Table 1. There was a high ratio of controls to cases used in the study to allow for the determination of the possible effects of rare SNPs. There was a significantly greater proportion of females in both disease groups, but the controls were significantly older (by 5.9–6.0 years).

### 3.2. AITD Genome-Wide Association Study

Details for genome-wide significant loci (*p* < 5 × 10^−8^), including the single-nucleotide polymorphism (SNP) with the lowest *p* value at each locus, are shown in Table 2.

**Figure 1:**
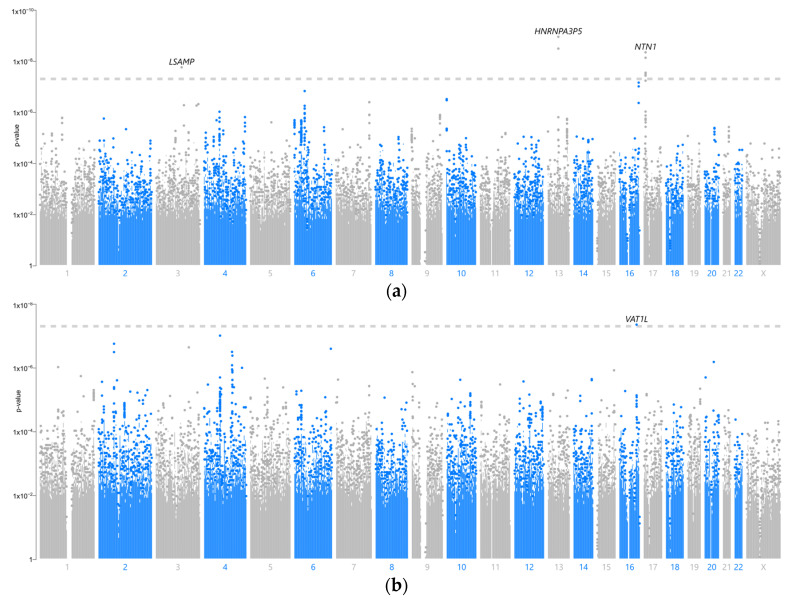
Manhattan plots showing the associations between SNPs and (**a**) Graves’ disease and (**b**) Hashimoto thyroiditis.

The overall results of the genome-wide association analyses are summarised in Figure 1. One locus on chromosome 16 in the VAT1L gene was significantly associated with HT, and three loci were significantly associated with GD (chromosomes 3, 13, and 17 in LSAMP, HNRNPA3P5, and NTN1, respectively). Most of the lead SNPs of significantly associated characteristics had low population minor allele frequencies (<5%) and a fairly large effect (OR > 3). However, similar to many GWASs, genes at these loci did not seem to be directly involved in autoimmune diseases based on the current knowledge of their functions.

#### Candidate Genes

**Figure 2:**
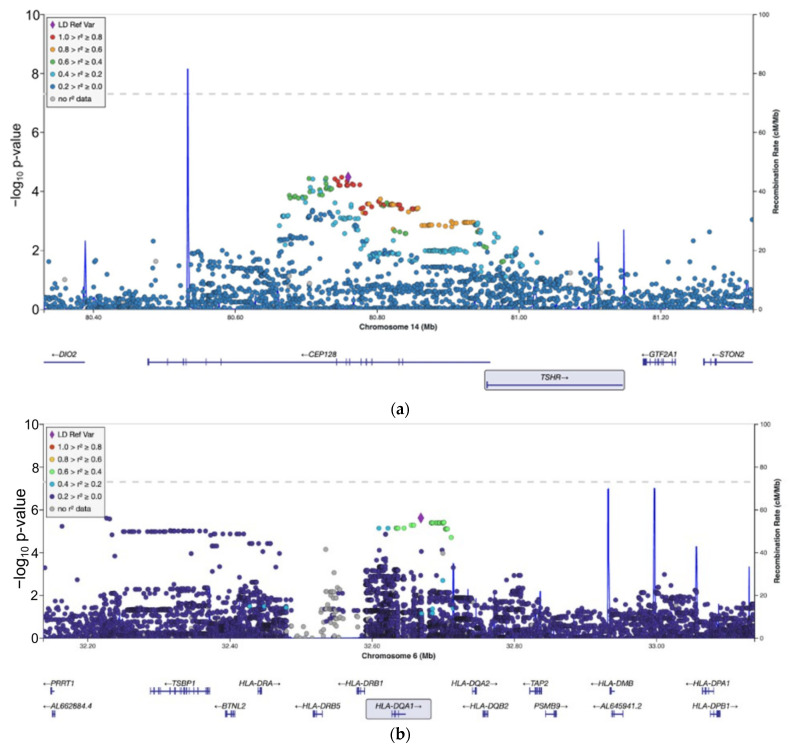
Regional association plots of (**a**) CEP128 and TSHR and (**b**) HLA-DQA1.

After a literature review, we selected 26 candidate genes (HLA-DQA1, CEP128, HLA-B, SELENOS, HLA-DRB1, HLA-DQB1, TG, SBNO2, TSHR, DMGDH, IL2RA, GPX4, TXNRD1, CCDC152, LOC105370596, PTPN22, CD40, SELENOP, LOC101928462, HLA-DRB3, FCRL3, FOXP3, CTLA4, RHOA, GPX3, and GPX1) with 5656 variants in our imputed dataset. After multiple testing corrections, we observed one significant cluster of associations with GD in the HLA-DQA1 gene, specifically the polymorphisms rs6933289 (*p* = 7 × 10^−5^, OR = 2.8 (1.8–4.5)) and rs6932167 (*p* = 7 × 10^−5^, OR = 2.8 (1.8–4.5)). The SNP cluster including the CEP128 and TSHR genes had a *p* value of 3 × 10^−4^ before multiple correction. Regional plots for each significant locus are shown in Figure 2.

### 3.3. Associations with the Serum Selenium Concentration (Case–Control Design)

The characteristics of the serum selenium GWAS participants are listed in Table 3. In this part of the study, 81 controls, 72 GD and 146 HT patients were analysed. Male and female proportions were comparable between study groups, but the control group participants were significantly younger than patients with GD or HT. Selenium levels were significantly lower in the GD group than in the control group.

**Figure 3:**
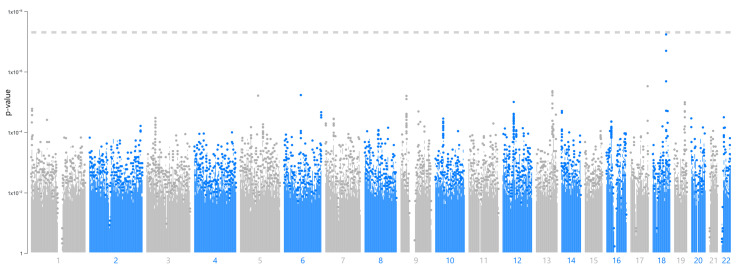
Manhattan plot showing the association between the SNP rs6567243 and a serum selenium concentration > 80 µg/L.

**Figure 4:**
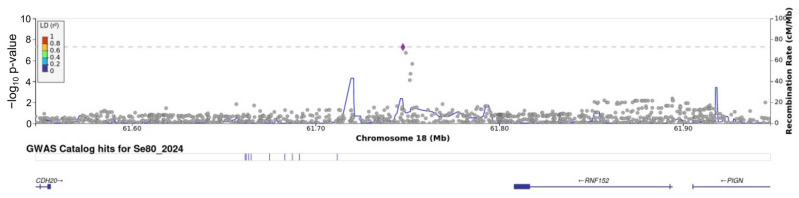
Detailed regional view of the leading associated SNP rs6567243.

A GWAS for a serum selenium concentration > 80 µg/L identified the significant SNP rs6567243 on chromosome 18 in our study population. This SNP has a high minor allele frequency in the non-Finnish European reference population (Table 4). A Manhattan plot is shown in Figure 3, and a regional plot for this locus is shown in Figure 4.

#### Selenium-Level Candidate SNPs

After a literature review, we identified the previously described SNPs on chromosome 5 (rs921943, rs17823744) and chromosome 21 (regions around rs6586282, rs1789953, rs234709) associated with selenium that were checked in our sample. None of these SNPs were significantly associated with a dichotomous serum selenium concentration > 80 µg/L in our population.

The previously described SNP rs921943 on chromosome 5 showed a trend towards increased linear serum selenium levels in our study population: selenium levels were greater in individuals carrying the C allele of rs921943. Both wild-type homozygotes and heterozygotes of chromosome 5, rs17823744, had virtually the same serum selenium level, while two alternate homozygotes had lower (but not statistically significant) levels.

SNPs associated with an increased serum selenium level in dichotomous analysis (chromosome 18, rs6567243) in the Latvian population also showed a significant association with the quantitative selenium level (beta = 10.2, *p* = 3.1 × 10^−6^) and quite clear additive allele dosage (Table 5).

SNPs from chromosome 21 were not available on our array and not imputed; therefore, no analysis with selenium levels was possible.

## 4. Discussion

Our GWAS revealed an association between GD and three significant loci, *LSAMP*, *NTN1*, and *HNRNPA3P5*, which have not been related to AITD to date. The encoded preprotein of LSAMP is processed by proteolysis and forms a neuronal surface glycoprotein. This protein might play an important role in neuronal growth and axon guidance during the development of the limbic system. Moreover, the preprotein of LSAMP has been proposed to have tumour suppressor properties and a role in neuropsychiatric disorders [22]. In other studies, changes in *LSAMP* have been associated with Meniere’s disease and decreased bone mineral density [23,24].

In addition, we found that *NTN1* (netrin-1) was associated with GD. A high serum netrin-1 concentration lowers the risk of ischaemic stroke and is negatively associated with outcomes after ischaemic stroke [25]. *NTN1* has been proposed as a potential candidate gene for Parkinson’s disease treatment [26]. Interestingly, a variant of *NTN1* (rs4791331) has been associated with an increased risk of left cleft lip with or without cleft palate [27]. In functional zebrafish studies, netrin-1 was shown to potentially contribute to aberrant cardiovascular development and thyroid dysgenesis [28].

We also found an association between GD and heterogenous nuclear ribonucleoprotein A3 pseudogene 5 (*HNRNPA3P5*) that has not been previously described.

As suggested by candidate gene studies, HT-susceptible genes are mainly associated with the immune response (*HLA*, *CTLA4*, *IL1RN*, *IL1β*, *IL17F*, *GITR*, *and STAT3*). However, these findings are not sufficient due to a lack of adequately powered studies and replication [29]. We identified only vesicle amine transport 1-like (*VATL1*) as a candidate gene locus; this gene has been previously described in association with spinocerebellar ataxia but not in relation to AITD [30].

Overall, the previously described functions of these candidate genes are not clearly related to AITD phenotypes, and several of these genes are poorly characterised in genetic databases and the current literature. The role of these novel loci in physiological functions and disease pathogenesis is ambiguous; therefore, additional knowledge about these candidates is valuable. This study could promote further in-depth investigations of the functional role of these genes at the molecular level, and their relationship with AITD could be assessed more thoroughly.

In our study cohort, we also assessed previously identified loci as “candidate genes” and detected significant associations between *HLA-DQA1*, *CEP128* and *TSHR* in a limited pool of genetic markers; however, none of the associations were significant after multiple testing corrections at the genome-wide level. The lack of previously confirmed associations, especially at HLA loci, might be attributed to the variability in the HLA locus, which could not be fully investigated in such a small sample. Moreover, the high ORs could indicate relatively rare subgroups of GD and HT patients, which can be highlighted by the use of a large control cohort.

In addition to genetic factors, micronutrients such as iodine and selenium have been associated with AITD and thyroid dysfunction. Selenium is an essential micronutrient present in several proteins. In adults, the largest amount of selenium per gram of tissue is found in the thyroid in the form of selenoproteins [31], which are necessary for thyroid hormone metabolism and proper antioxidant function. Low levels of selenium are related to a higher incidence of AITD [32,33]. Consistent with our findings, other studies have reported lower selenium levels in patients with GD [34,35].

In turn, selenium supplementation in patients with AITD, particularly those with HT, reduces anti-TPO levels, improves quality of life, and delays disease progression in patients with Graves’ orbitopathy [11,33].

Selenium is found in nutritional products as selenomethionine, selenocysteine, selenate or selenite [36]. Most of the selenium in the serum is present in the form of selenocysteine in selenoprotein P (SePP) and glutathione peroxidase 3 (GPx3), and the rest is bound to albumin and other proteins, such as selenomethionine [37]. Selenium concentrations are affected by several factors, such as sex, age, BMI, smoking habits, and socioeconomic status [38]. Candidate gene studies have suggested that selenium levels also depend on genetic variations [39].

Previous studies have confirmed the significant association between selenium levels and genetic variations in dimethylglycine dehydrogenase (*DMGDH*, encoding DMGDH) [40,41]. *DMGDH* rs921943 is also associated with higher baseline selenium concentrations in pregnant women, although this SNP does not affect selenium levels throughout pregnancy or after supplementation [38]. We observed a trend towards an increase in the serum selenium concentration associated with this SNP in our study population. An association was shown by the novel SNP rs6567243 with serum selenium levels; individuals carrying the G allele presented significantly higher serum selenium levels, suggesting important involvement and necessity to have further studies of this SNP and locus with selenium levels.

The SNP rs6567243 is found on chromosome 18 and encodes *LINC01544/RNF152/PIGN*. *LINC01544* is a nonprotein coding RNA gene. RNF152 controls mammalian targets of rapamycin complex 1 (mTORC1) signalling, contributes to the reaction of cells to amino acid starvation [42], and facilitates the polyubiquitination of specific proteins, leading to their subsequent transportation to the proteasome for breakdown [43]. *RNF152* is associated with breast, prostate, and colorectal cancer [44]. Data from observational studies analysing selenium exposure and cancer risk indicate a reduced risk of developing specific cancers at particular sites, including stomach, colorectal, lung, breast, bladder, and prostate cancers; however, further investigation is needed to evaluate whether selenium might alter the likelihood of cancer in individuals with specific genetic profiles or nutritional statuses [45].

*PIGN* encodes a protein involved in the biosynthesis of glycosylphosphatidylinositol (GPI) anchors that play a crucial role in tethering proteins to the cell surface [46]. Downregulation of the *PIGN* gene has been described in keloid fibroblasts after exposure to selenocysteine [47].

The clinical implications of the study findings could be further explored, particularly in light of the impact of genetic factors on the frequent co-occurrence of AITD with other autoimmune conditions. As established, a combination of genetic, immune, environmental, and dietary factors significantly contributes to the development of AITD. Notably, AITD is commonly observed in both adult and pediatric patients with celiac disease. A previous study [48] aimed to assess the prevalence of coeliac disease among individuals with autoimmune thyroid dysfunction through serological screening for gluten-sensitive enteropathy. Sera from 220 patients diagnosed with autoimmune thyroiditis, 50 euthyroid subjects presenting with thyroid nodules, and 250 healthy blood donors were subjected to examination for IgA anti-tissue transglutaminase (anti-tTG) and antiendomysial antibodies (EmA). The findings revealed that among patients with autoimmune thyroiditis, seven individuals tested positive for IgA anti-tTG, while IgA EmA positivity was detected in only six cases. Subsequent duodenal biopsy confirmed coeliac disease in seven patients, with varying degrees of villous atrophy observed. Interestingly, the majority of coeliac patients exhibited no clinical manifestations of malabsorption. Comparatively, all euthyroid controls demonstrated negative IgA antibody results, with a solitary blood donor exhibiting dual positivity for IgA anti-tTG and EmA, indicative of coeliac disease. The prevalence of coeliac disease in autoimmune thyroiditis patients (3.2%) significantly exceeded that observed in blood donors (0.4%).

AITD is also evident within the spectrum of autoimmune polyendocrine syndromes. A study by Pallotta et al. [49] emphasizes the intricate relationship between celiac disease (CD) and thyroid autoimmunity within the broader context of autoimmune polyglandular syndromes (APS). Notably, the prevalence of APS in Italian CD outpatients was found to be 1.4%, with the majority belonging to APS type 3 (defined as AITD plus another autoimmune disorder in the absence of Addison disease). This finding underscores the necessity of thorough screening for thyroid disorders, such as autoimmune thyroiditis, in CD patients due to their heightened susceptibility to endocrine autoimmunity.

The association with celiac disease may serve as an illustrative example wherein genetic factors and micronutrient deficiencies, such as reduced levels of iodine and selenium due to malabsorption, are presumed to significantly contribute to the frequent co-occurrence of these disorders.

Moreover, genetic factors may contribute to the observed variations in the age of onset of AITDs among individuals with other autoimmune disorders. Previous studies have demonstrated that in patients with autoimmune hepatitis (AIH), a condition often associated with AITD, elderly AIH patients exhibit distinct characteristics compared to younger cohorts, including a notably higher prevalence of concomitant AITD and HLA-DR4 expression [50]. This observation suggests a potentially significant role of genetic factors in shaping regional disparities in the epidemiology of AITD and warrants further investigation.

## 5. Conclusions

We identified three novel loci associated with GD (*LSAMP*, *HNRNPA3P5*, and *NTN1*), one associated with HT (*VAT1L*), and one associated with a serum selenium concentration >80 µg/L (*LINC01544/RNF152/PIGN*), which also contributes to the additive increase in serum selenium in the Latvian population. The potential functional role of these novel loci needs to be further studied via genetic and cellular experiments to determine their specific impact on the pathogenesis of AITD. Whether the association could be attributed to population-specific effects or unknown stratification in our cohort is unclear, and further assessment of these findings is needed to explain the relationships of genetic traits with AITD and other phenotypes.

## Figures and Tables

**Table 1:** Characteristics of the genotyped cohort of AITD GWAS.

Parameter	Controls (*n* = 2442)	GD (*n* = 148)	HT (*n* = 102)	*p* Values (Control-GD and Control-HT)
Sex				
Female	1477 (60.5%)	122 (82.4%)	85 (83.3%)	<0.0001 both
Male	965 (39.5%)	26 (17.6%)	17 (16.7%)	<0.0001 both
Age				
Mean (SD)	54.3 (14.0)	48.4 (15.6)	48.3 (15.6)	<0.0001 both
Median [Min, Max]	55.0 [18.0, 94.0]	50.5 [21.0, 84.0]	52.0 [22.0, 78.0]	

ns—not significant, SD—standard deviation; GD—Graves’ disease.

**Table 2:** Genotype association with thyroid disease status in retrospective study (only lead SNP of locus reported).

Tested Phenotype	Pos	Alleles	Freq% *	Cases/ Controls	Lead SNP ID	Nearest Gene	OR (CI 95%)	*p* Value
GD	chr 13: 65 718 934	T/C	4.4	148/2442	rs117860697	*HNRNPA3P5*	3.3 (2.2–4.6)	1.13 × 10^−9^
GD	chr 17: 9 016 416	A/G	0.5	148/2442	rs189272113	*NTN1*	6.9 (3.6–13.1)	4.55 × 10^−9^
GD	chr 3: 117 084 612	T/C	0.9	148/2442	rs147639537	*LSAMP*	7.0 (3.5–13.7)	1.76 × 10^−8^
HT	chr 16: 77 939 442	A/C	3.1	102/2442	rs7184775	*VAT1L*	4.9 (2.8–8.7)	4.45 × 10^−8^

* Non-Finnish European population frequency, chr—chromosome, freq—frequency, SNP ID—single-nucleotide polymorphism identifier, OR—odds ratio; CI—confidence interval.

**Table 3:** Characteristics of the genotyped cohort of serum selenium GWAS participants.

Parameter		Controls (*n* = 81)	GD (*n* = 72 *)	HT (*n* = 146 *)	*p* Value	
					Control-GD	Control-HT
Sex	Males	12	8	15	0.53	0.32
	Females	69	63	131		
Mean age ± SD		37.4 ± 13.1	49.6 ± 15.5	50.7 ± 15.9	<0.0001	<0.0001
Median age ± SD (range)		32 (22–72)	53 (21–81)	51.5 (18–78)	-	
Mean serum Se ± SD		88.2 ± 33.7	71.8 ± 19.4	95.2 ± 21.9	0.0004	0.06
Median serum Se (IQR)		83.4 (69.0–115.8)	69.4 (68.0–106.2)	93.7 (80.4–110.4)	-	

*—one participant had a history of both GD and HT, SD—standard deviation, GD—Graves’ disease, HT—Hashimoto thyroiditis; IQR—interquartile range.

**Table 4:** Genotype association with a serum selenium concentration > 80 µg/L.

Tested Phenotype	Pos	Alleles	Freq% *	Cases/ Controls	Lead SNP ID	Nearest Gene	OR (CI 95%)	*p* Value
Serum selenium > 80 µg/L	chr 18: 61 747 557	G/**A**	54.8	169/128	rs6567243	LINC01544/ RNF152/PIGN	3.24 (2.1–4.9)	5.31 × 10^−8^

* Non-Finnish European population frequency, chr—chromosome, freq—frequency, OR—odds ratio; CI—confidence interval.

**Table 5:** Serum selenium concentrations (µg/L) by genotype.

SNP	G11	G12	G22
Chr5 rs921943			
Genotype	T/T	T/C	C/C
Count (*n* = 297)	33	145	119
Serum selenium, mean (SD)	83.0 (31.1)	87.0 (25.8)	90.1 (26.7)
Chr5 rs17823744			
Genotype	G/G	G/A	A/A
Count (*n* = 297)	2	77	218
Serum selenium, mean (SD)	60.0 (2.6)	88.0 (28.4)	88.0 (26.2)
Chr18 rs6567243			
Genotype	G/G	G/A	A/A
Count (*n* = 297)	71	153	73
Serum selenium, mean (SD)	96.1 (24.3)	89.7 (27.4)	75.7 (23.6)

SNP—single-nucleotide polymorphism, G—genotype, chr—chromosome; SD—standard deviation.

## Data Availability

The datasets used and/or analysed during the current study are available from the corresponding author upon reasonable request.
